# Anosmia and Ageusia as the Only Indicators of Coronavirus Disease 2019 (COVID-19)

**DOI:** 10.7759/cureus.7918

**Published:** 2020-05-01

**Authors:** Qian Zhang, Khine S Shan, Shahrzad Abdollahi, Travis Nace

**Affiliations:** 1 Internal Medicine, Abington Hospital-Jefferson Health, Abington, USA; 2 Internal Medicine, University of Maryland Medical Center, Baltimore, USA; 3 Library Science, Abington Hospital-Jefferson Health, Abington, USA

**Keywords:** anosmia, loss of smell, covid-19, novel coronavirus, coronavirus, atypical coronavirus, ageusia, loss of taste

## Abstract

The patient is a 60-year-old woman with a history of vertigo and seasonal allergies who presented to the hospital with the chief complaint of headache. Radiological findings were negative for intracranial abnormalities. The headache was due to trigeminal neuralgia. She had concurrent complaints of anosmia and ageusia without fever, respiratory symptoms, or obvious risk factors. However, it was determined to test the patient for coronavirus disease 2019 (COVID-19) infection despite extremely low clinical suspicion. Unfortunately, she was found to be COVID-19 positive after she was discharged from the hospital while she remained asymptomatic. There is currently a lack of published case reports describing COVID-19 patients with the sole symptoms of anosmia and ageusia in the United States of America.

## Introduction

COVID-19, or coronavirus disease 2019, originated from Wuhan, China, in December 2019. It is caused by novel enveloped single-stranded ribonucleic acid (RNA) betacoronavirus, which is known as the severe acute respiratory syndrome coronavirus 2 (SARS-CoV-2) [1]. This disease has since quickly spread worldwide within a few months. The World Health Organization (WHO) reports that there are a total of 1,353,361 global cases along with 79,235 total deaths as of April 9, 2020 [2]. Typical symptoms include fever, cough, and shortness of breath [3]. While these symptoms are the typical presentations, other symptoms are gaining more attention as possible indicators of the disease as our understanding of the disease is rapidly evolving. These atypical symptoms include olfactory and gustatory dysfunctions [4]. There is a lack of evidence in the current medical literature of anosmia or ageusia in patients suspected of having COVID-19 infection. The Center for Disease Control (CDC) currently does not include these findings as symptoms of COVID-19 but does state that medical awareness should be increased in this setting [3]. Likewise, the WHO does not include these symptoms as part of the differential diagnosis but does mention that other unconventional symptoms, such as myalgia, nasal congestion, runny nose, sore throat or diarrhea, do exist [5]. Physicians are sounding the alarm to this lack of attention and evidence in the medical literature. This case report details a 60-year-old woman with the chief complaint of right-sided headache along with anosmia and ageusia but was eventually found to be SARS-COV-2 positive.

## Case presentation

Our patient is a 60-year-old woman who presented to the emergency department (ED) with the chief complaint of right-sided headache for the past week. She had a past medical history of asthma, vertigo, seasonal allergies, and anxiety. Her headache was located predominantly at the right temporal, retro-ocular, and retro-auricular regions. She described the headache as an "electrical sensation" that was associated with occasional nausea, blurry vision in both eyes accompanied by a metallic taste in the mouth with a loss of smell sensation. She denied any fever, sore throat, rhinorrhea, cough, myalgia, vomiting, dysphagia, neck pain, chest pain, shortness of breath, weakness, or sensory disturbances. The review of systems was otherwise unremarkable. She had magnetic resonance imaging (MRI) in the outpatient setting last year due to vertigo and the result was unremarkable. She was generally healthy otherwise and did not take any medications except for occasional loratadine for seasonal allergies. She is currently retired, lives at home, denies any smoking history or recent traveling. She was recommended by her primary care physician (PCP) to take amoxicillin to treat for sinusitis as the possible underlying cause of her symptoms. However, she elected to defer the treatment but rather decided to present to the ED for further evaluation.

**Figure 1:**
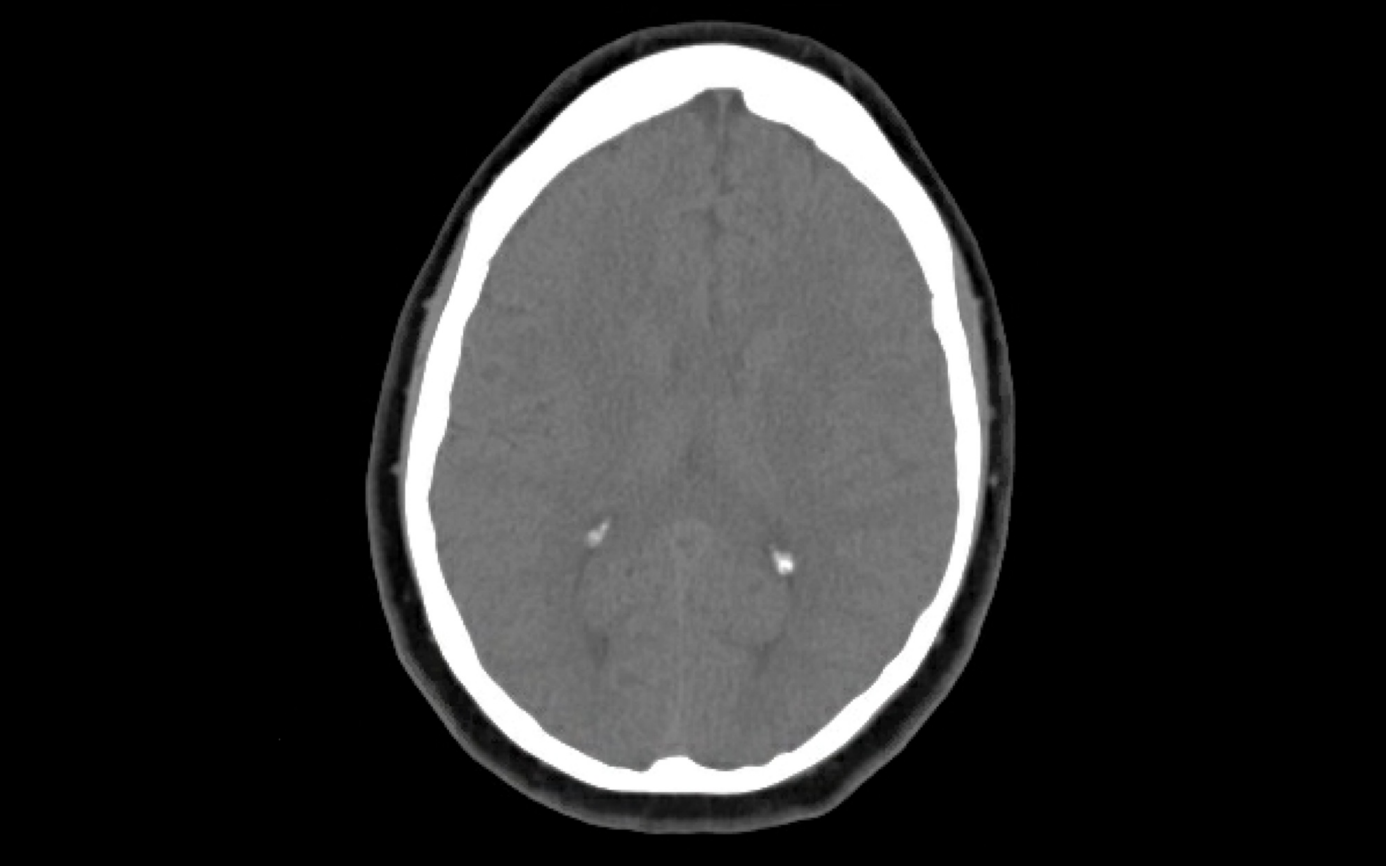
CT head without contrast No acute intracranial hemorrhage, extracerebral fluids collection, acute transcortical infarction, midline shift, or mass effect CT: computed tomography

In the ED, her initial vital signs were: temperature 98.1°F, blood pressure 142/81 mmHg, respiratory rate 18 breaths per minute, heart rate 84 beats per minute, and oxygen saturation 95% on ambient air. Physical examination was completely unremarkable. The only abnormal laboratory results were: erythrocyte sedimentation rate (ESR) 31 mm/hr, alanine transaminase (ALT) 31 U/L. Computed tomography (CT) head without contrast showed no acute intracranial abnormality (Figure 1). Her headache was thought to be related to trigeminal neuralgia and was prescribed Gabapentin 300 mg twice a day. There were also concerns for possible COVID-19 infection due to anosmia and ageusia but additional testing was deferred due to the lack of fever, respiratory distress, and laboratory abnormalities. This decision was made upon extensive discussion with the infectious disease specialist, as the clinical suspicion for COVID-19 was extremely low because it could be related to her seasonal allergies. The patient remained stable overnight.

**Figure 2:**
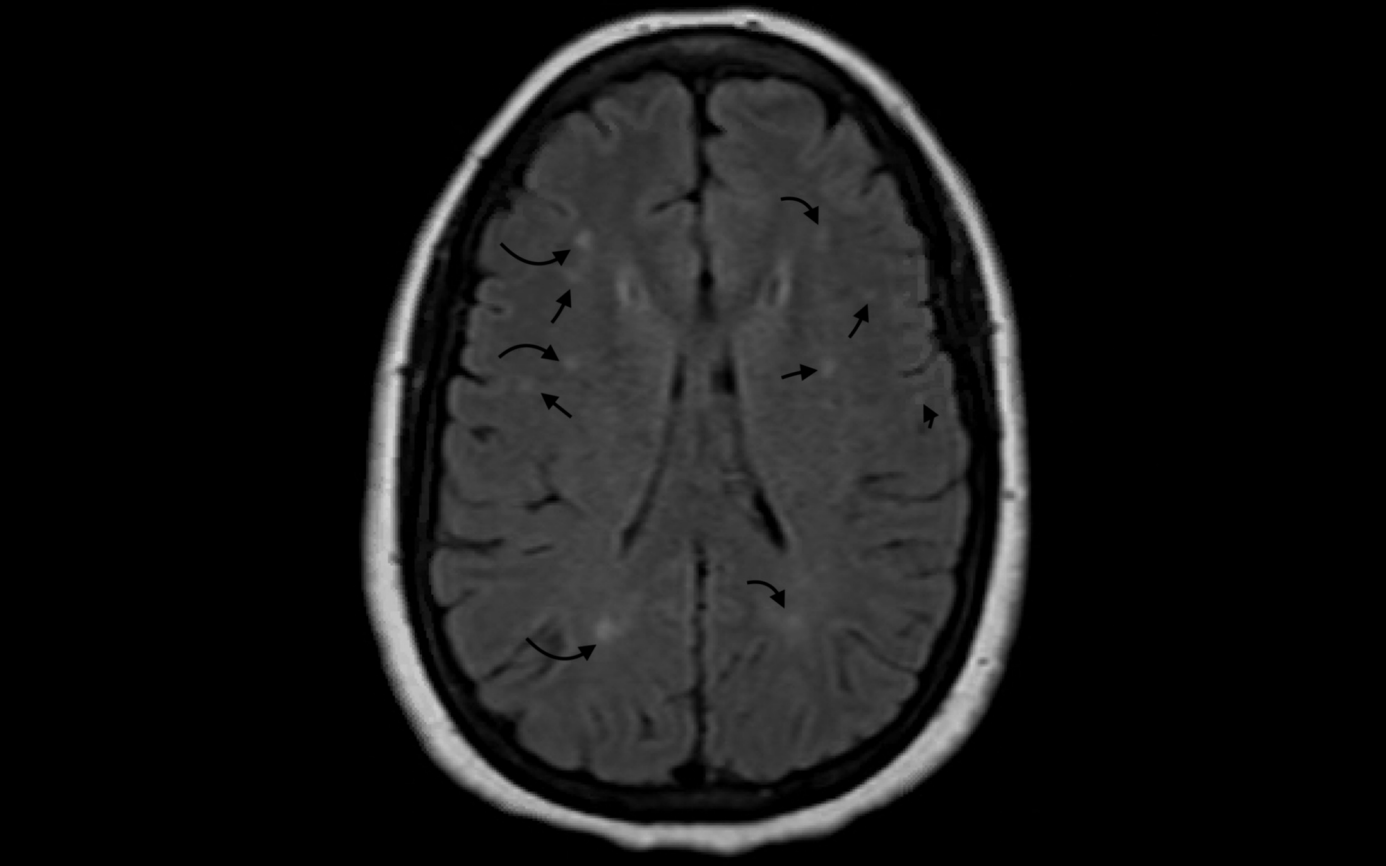
MRI of the brain Stable extensive white matter changes without the presence of acute infarction MRI: magnetic resonance imaging

Her headache improved on Days 2-3 of hospitalization. MRI of the brain found stable extensive white matter changes without acute ischemic findings (Figure 2). She was eventually placed in airborne isolation and SARS-COV-2 was sent to rule out COVID-19 infection despite low clinical suspicion. She was subsequently discharged from the hospital and was instructed to self-quarantine given pending results. SARS-COV-2 came back to be positive after one day. She was notified over the phone and was recommended to continue to self-quarantine and seek medication attention if there are new symptoms.

## Discussion

The most common COVID-19 symptoms include fever (43.8% on initial presentation and 88.7% during hospitalization), cough (67.8%), nasal congestion (4.8%), nausea or vomiting (5.0%), and diarrhea (3.8%) based on a research study of 1099 patients from China. However, both anosmia or ageusia were not mentioned as both common and atypical symptoms in this study. Significant laboratory findings that were listed include lymphocytopenia, thrombocytopenia, leukopenia, elevated C-reactive protein, creatinine kinase, and d-dimer [1]. However, there are other, newly emerging initial presenting symptoms that were absent in the Chinese patients, as COVID-19 spreads quickly across the globe. They include asymptomatic carriers and other nonspecific symptoms that do not generally arouse suspicion of a possible COVID-19 infection.

Most hospitals in the United States utilize COVID-19 screening criteria that include cough, fever, and/or respiratory distress. Unfortunately, these screening criteria may miss out on silent carriers and those with atypical presentations. Furthermore, Italy and France surprisingly reported a large population of patients with symptoms of anosmia and/or ageusia in a COVID-19 confirmed diagnosis. In a letter to the editor submitted to the journal Obesity, Jean-François Gautier and Yann Ravussin stated that they have observed cases of anosmia in patients following two to three days of fatigue and headache [4]. Epidemiologist Hendrik Streek mentioned that two-thirds of the patients have anosmia and ageusia lasting several days in Germany [6]. In addition, 30% of COVID-19 confirmed cases in South Korea have had the primary initial symptom of anosmia [4]. One preliminary study from China by Mao et al. reported that 5.1% of the patients with COVID-19 had anosmia and 5.6% had ageusia [7]. In another study of 320 patients from Italy by Vaira et al., 19.4% of patients had chemosensory dysfunction not associated with rhinitis or nasal obstruction. However, these data have been underestimated as symptoms of anosmia and ageusia were not always addressed [8].

Our patient had a very low clinical suspicion of COVID-19 infection, as she was afebrile along with no respiratory symptoms despite having anosmia and ageusia in the setting of headache caused by trigeminal neuralgia. Fortunately, she was adequately tested given that cases of atypical presentation have been reported in the past. Gane et al. described a similar case report of a patient with positive COVID-19 who presented with isolated anosmia but was otherwise asymptomatic [9].

There are a few hypothesized mechanisms of action on why COVID-19 patients may develop anosmia and ageusia despite their association with SARS-CoV-2 not having been yet established. It could be due to direct damage of the virus on olfactory and gustatory receptors [8]. The nasal epithelium contains olfactory epithelium (OE) and olfactory sensory neurons (OSNs). OE contains basal stem cells that are responsible for renewing sustentacular cells and OSNs. It also contains microvillar cells and mucus-secreting Bowman’s gland cells. Sustentacular cells structurally support sensory neurons, detoxify, and maintain the salt and water balance. It is thought that SARS-CoV-2 infects cells through interaction between its spike (S) protein and the angiotensin-converting enzyme 2 (ACE2) protein on target cells. This interaction requires cleavage of the S protein by the cell surface protease TMPRSS2. Therefore, ACE2 and TMPRSS2 are required for SARS-CoV-2 to infect cells [10]. The study of mouse and human RNA sequencing datasets by Brann et al. showed that OE expresses two key genes required for SARS-CoV-2 entry: ACE2 and TMPRSS2. OSNs, on the contrary, did not show any gene expression. Sustentacular cells in OE express these genes at levels comparable to those found in lung cells. Thus, it suggests that SARS-CoV-2 may infect OE that contains sustentacular cells, leading to damage of OE and disturbing the function of OSN. Loss of sustentacular cells and the inability to regenerate OE over time can result in long-lasting anosmia. In addition, damage to microvillar cells in OE might alter iron gradients and thus affect the function of sensory neurons. Damage to the Bowman’s gland cells could cause disruption of the olfactory neuroepithelium. However, due to the relatively new identification of SARS-CoV-2-associated anosmia, no formal experiments have been performed to explore SARS-CoV-2’s influence on OE. Moreover, it is unclear whether the olfactory abnormality is due to dysfunction in the higher-order olfactory structures. It has shown previously that viruses including coronavirus can propagate to olfactory bulb or piriform complex even though the exact mechanisms of action are unknown. It is also unclear whether the impact of SARS-CoV-2 on smell is responsible for the alteration in taste perception [10].

Even though the association of anosmia with SARS-CoV-2 is currently not well-known, previous respiratory viruses, such as coronaviruses, have shown to cause problems with smell receptors and have been associated with post-viral anosmia [11]. A very recent multicenter European study reported that 11.8% of patients with SARS-CoV-2 presented with olfactory dysfunction prior to other symptoms. There was also a high prevalence (around 85%) of olfactory or gustatory dysfunction among those patients with SARS-CoV-2 [12]. 

Thus, further research on the prevalence, duration, and severity of anosmia and ageusia in patients with COVID-19 is needed, as it is important to guide the prompt diagnosis, treatment, and prevention of COVID-19 in the setting of an ongoing pandemic.

## Conclusions

The understanding of COVID-19 is rapidly evolving, as we are in the middle of an unforeseen, ongoing pandemic. Symptoms of COVID-19 patients range widely from fever, respiratory symptoms, to newly reported findings of anosmia and ageusia from South Korea, Italy, and France. Awareness of a possible COVID-19 infection should be raised in patients with the sole presentation of anosmia and ageusia despite the lack of published case reports or research findings on its exact mechanisms of action. Extra attention is sometimes a game-changer for patient care and safety especially as we are situated in uncharted territory. COVID-19 discoveries are being made every day and healthcare providers should closely follow its footsteps to ensure the best care is delivered to all patients.
